# Novel thermostable GH5_34 arabinoxylanase with an atypical CBM6 displays activity on oat fiber xylan for prebiotic production

**DOI:** 10.1093/glycob/cwac080

**Published:** 2022-12-06

**Authors:** Siri Norlander, Andrius Jasilionis, Zubaida Gulshan Kazi Ara, Carl Grey, Patrick Adlercreutz, Eva Nordberg Karlsson

**Affiliations:** Division of Biotechnology, Department of Chemistry, Lund University, PO Box 124, SE-221 00 Lund, Sweden; Division of Biotechnology, Department of Chemistry, Lund University, PO Box 124, SE-221 00 Lund, Sweden; Division of Biotechnology, Department of Chemistry, Lund University, PO Box 124, SE-221 00 Lund, Sweden; Division of Biotechnology, Department of Chemistry, Lund University, PO Box 124, SE-221 00 Lund, Sweden; Division of Biotechnology, Department of Chemistry, Lund University, PO Box 124, SE-221 00 Lund, Sweden; Division of Biotechnology, Department of Chemistry, Lund University, PO Box 124, SE-221 00 Lund, Sweden

**Keywords:** arabinoxylanase, arabinoxylo-oligosaccharides, carbohydrate binding module, enzyme characterization, homology modeling

## Abstract

Carbohydrate active enzymes are valuable tools in cereal processing to valorize underutilized side streams. By solubilizing hemicellulose and modifying the fiber structure, novel food products with increased nutritional value can be created. In this study, a novel GH5_34 subfamily arabinoxylanase from *Herbinix hemicellulosilytica*, *Hh*Xyn5A, was identified, produced and extensively characterized, for the intended exploitation in cereal processing to solubilize potential prebiotic fibers: arabinoxylo-oligosaccharides. The purified two-domain *Hh*Xyn5A (catalytic domain and CBM6) demonstrated high storage stability, showed a melting temperature *T*_m_ of 61°C and optimum reaction conditions were determined to 55°C and pH 6.5 on wheat arabinoxylan. *Hh*Xyn5A demonstrated activity on various commercial cereal arabinoxylans and produced prebiotic AXOS, whereas the sole catalytic domain of *Hh*Xyn5A did not demonstrate detectable activity. *Hh*Xyn5A demonstrated no side activity on oat β-glucan. In contrast to the commercially available homolog *Ct*Xyn5A, *Hh*Xyn5A gave a more specific HPAEC–PAD oligosaccharide product profile when using wheat arabinoxylan and alkali extracted oat bran fibers as the substrate. Results from multiple sequence alignment of GH5_34 enzymes, homology modeling of *Hh*Xyn5A and docking simulations with ligands XXXA^3^, XXXA^3^XX and X^5^ concluded that the active site of *Hh*Xyl5A catalytic domain is highly conserved and can accommodate both shorter and longer ligands. However, significant structural dissimilarities between *Hh*Xyn5A and *Ct*Xyn5A in the binding cleft of CBM6, due to the lack of important ligand-interacting residues, is suggested to cause the observed differences in substrate specificity and product formation.

## Introduction

Enzymatic processing of biomass is a widespread approach and an important step in biorefineries and food industry, in order to break down recalcitrant structures and valorize individual process streams to various building-block chemicals, speciality chemicals and consumer products (Silva et al. 2018; Ostby et al. 2020). Carbohydrate acting enzymes such as glycoside hydrolases (GH) is one example of an essential group of enzymes used on forest and agricultural materials for this purpose, employed to hydrolyze carbohydrates into shorter oligosaccharides and monosugars (Gilbert et al. 2008). Endo-β-1,4-xylanases (EC 3.2.1.8) catalyze the cleavage of β-1,4 linkages in the backbone of xylans, their activity varying depending on the type and extent of substitutions on the xylan and can be found in GH families such as GH10, GH11, GH30 and GH5. Xylanases that specifically cleave the hemicellulose arabinoxylan (AX) present in many plants are especially important for valorizing fiber-rich side-streams from processing of cereals and grains containing high amounts of AX (Nordberg Karlsson et al. 2018). Promising end products from the enzymatic hydrolysis of AX are xylo-oligosaccharides (XOS) and arabinoxylo-oligosaccharides (AXOS), which have shown potential as prebiotics by selectively stimulating the growth of probiotic bacteria, for example, *Lactobacillus* and *Bifidobacterium* species (Sajib et al. 2018; Bhattacharya et al. 2020). The probiotic bacteria can, in turn, help to maintain a healthy gut and microbiota by producing short-chain fatty acids and reducing cancer cell proliferation (Broekaert et al. 2011).

Depending on the enzymes employed in the process, the resulting XOS and AXOS can have different lengths and substituent patterns, which are important aspects to consider when customizing the final product for stimulating selected bacterial species. Xylanases from family GH10 and GH11 are commonly used for AX degradation (Nordberg Karlsson et al. 2018). However, their activity and specificity vary substantially, depending on the biomass origin and substrate purity, as these enzymes can be more or less restricted by extensive substitution (Linares-Pasten et al. 2018; Schmitz et al. 2022b). In addition, xylanases from families GH10 and GH11 often display some side activity on mixed linkage β-glucan (Shi et al. 2010), a valuable fiber abundant in barley and oat. Oat β-glucans of high molecular weight have proven to be health beneficial by lowering blood glucose levels and cholesterol (Wang and Ellis 2014), explaining why the side activity could be undesired for certain cereal processes.

Family GH5 subfamily 34 (GH5_34) consists mainly of multimodular, AX-specific endo-β-1,4-xylanases, or arabinoxylanases (EC 3.2.1.-), which are reported to require an O3-linked arabinose substituent in the active site (in a subsite termed −2^*^, adjacent to the −1 subsite) to be able to cleave the xylan backbone (Correia et al. 2011; Labourel et al. 2016). These enzymes are also of interest because of their ability to accommodate several arabinose substituents in the active site (in the glycone as well as the aglycone subsites), thereby possessing the catalytic activity to hydrolyze highly decorated substrates, aiding in the complete degradation of recalcitrant fibers and yielding unique AXOS product profiles (Correia et al. 2011; Labourel et al. 2016; Falck et al. 2018; Schmitz et al. 2022b). Arabinoxylanases are a common part of the cellulosome, a multi-enzyme complex used by microorganism for extracellular degradation of plant cell walls. It is therefore typical for these enzymes to include a dockerin domain, to mediate stable integration of the enzyme, as well as different carbohydrate-binding modules (CBM) in addition to their catalytic domain, to be able to bind various hemicellulose substrates (Bayer et al. 2004).

The enzymes are attributed to GH5_34 subfamily based on the sequence as well as structural similarity and catalytic conservation, having two glutamic acids acting as the catalytic nucleophile and proton donor, respectively (Correia et al. 2011; Labourel et al. 2016). To date, only nine GH5_34 subfamily sequence entries are indexed in the Carbohydrate Active enZymes (CAZy) database (http://www.cazy.org; Lombard et al. 2013), and only four enzymes have been characterized. The extensively characterized enzyme *Ct*Xyn5A from *Acetivibrio thermocellus* (initially *Clostridium thermocellum*) (Tindall 2019) remain as the only structurally characterized GH5_34 enzyme (Brás et al. 2011; Correia et al. 2011; Labourel et al. 2016), and this enzyme is commercially available in analytical amounts. Three other GH5_34 enzymes have previously shown activity on AX from rye, wheat and corn (Labourel et al. 2016), but these candidates have not been studied in further detail. Therefore, it is of interest to prioritize and characterize novel GH5_34 enzymes, in order to extend the knowledge of this small GH5 subfamily and identify industrially relevant enzymes with unique specificities and suitable traits for production of AXOS from agricultural biomass.

In this study, a novel GH5_34 subfamily arabinoxylanase, here termed *Hh*Xyn5A, was identified. The gene for *Hh*Xyn5A was initially assigned as originating from *Herbinix hemicellulosilytica,* an anaerobic thermophilic cellulose-converting bacterium isolated from a biogas reactor metagenome (Koeck et al. 2015). *Hh*Xyn5A was here produced, purified and characterized based on stability and substrate specificity, at favorable reaction conditions for cleavage of AX. Several other hemicellulose degrading enzymes have previously been characterized from *H. hemicellulosilytica* (attributed to families GH10, GH11, GH43 and GH51) (Mechelke et al. 2017); however, the arabinoxylanase investigated here has previously been overlooked. Moreover, in this study, the product profile of *Hh*Xyn5A was compared to the previously characterized *Ct*Xyn5A (Labourel et al. 2016), in order to evaluate its potential use in biomass processing, and interesting differences were identified. To allow deeper investigation of interacting residues, a tertiary structure model was created for performing docking simulations with different AXOS ligands. This is the first GH5_34 arabinoxylanase to be investigated from *H. hemicellulosilytica*, and one of few GH5_34 enzymes to be extensively characterized.

## Results

### Cloning, expression and purification of *Hh*Xyn5A variants

**Fig. 1:**
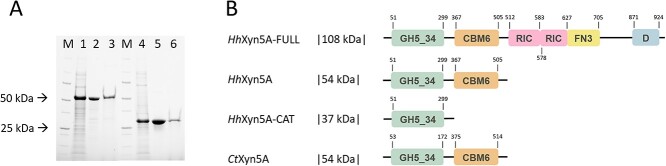
(**A**) SDS—PAGE of recombinant *Hh*Xyn5A variants. M—precision plus protein unstained standards (bio-rad) molecular-mass marker, (1) *Hh*Xyn5A in lysate before purification, (2) *Hh*Xyn5A after purification and dialysis, (3) *Hh*Xyn5A eluted in *Ct*Xyn5A formulation buffer, (4). *Hh*Xyn5A-CAT in lysate before purification, (5) *Hh*Xyn5A-CAT after purification and dialysis, (6) *Hh*Xyn5A-CAT eluted in *Ct*Xyn5A formulation buffer. The molecular weight for *Hh*Xyn5A and *Hh*Xyn5A-CAT variants is 54 and 37 kDa, respectively. (**B**) Schematic representation of the domain organization of the different enzyme variants of *Hh*Xyn5A and *Ct*Xyn5A. GH = glycoside hydrolase; CBM = carbohydrate binding module; RIC = ricin-type beta-trefoil lectin domain; FN3 = fibronectin type 3 like domain; D = dockerin domain. Numbering above the domains refer to the amino acid sequence interval.

Production of the full-length multi-modular enzyme *Hh*Xyn5A-FULL variant was not successful (data not shown), whereas the production and purification of the truncated *Hh*Xyn5A variants (Figure 1A, including the two-domain variant *Hh*Xyn5A (54 kDa) and the sole domain variant *Hh*Xyn5A-CAT (37 kDa)) resulted in high yields (0.15 and 0.23 g/L culture for *Hh*Xyl5A and *Hh*Xyl5A-CAT, respectively) of soluble recombinant proteins (Figure 1B). A single-step purification by immobilized metal ion affinity chromatography (IMAC) resulted in purity near to homogeneity of both *Hh*Xyn5A variants (Figure 1B). The purified enzyme variants were stably soluble even after storage for 3 months at 4°C, both in elution and reaction buffers.

**Table 1:** Melting temperatures (*T*_m_) ± standard deviation of the enzymes at different pH determined by the nanoscale differential scanning fluorimetry. Measurements were performed in triplicates and *T*_m_ was extracted from the first derivative of the absorbance ratio 350/330 nm.

pH	*Ct*Xyn5A	*Hh*Xyn5A	*Hh*Xyn5A-CAT
	*Melting temperature (T*_m*,*_°C*)*		
4	66.9 ± 2.0	53.0 ± 5.8	55.9 ± 0.6
5.5	67.6 ± 2.5	58.2 ± 0.4	63.3 ± 0.4
6.5	73.5 ± 0.2	61.0 ± 0.2	63.6 ± 0.1
8.1	64.6 ± 0.4	58.1 ± 0.1	63.8 ± 0.2
9	74.5 ± 0.1	58.1 ± 0.0	64.0 ± 0.3
6.5 and 1% (w/v) WAX	74.3 ± 0.3	64.2 ± 0.1	ND

ND = not determined; WAX = wheat arabinoxylan

**Table 2:** Substrate specificity of *Hh*Xyn5A and *Ct*Xyn5A determined in U/mg ± standard deviation of triplicate experiments using the di-nitrosalicylic acid assay. For each substrate, specific activity was determined after 10 min and 1 h, and total reducing end (red. ends) formation was determined after 24 h.

Substrate	*Hh*Xyn5A				*Ct*Xyn5A			
	10 min U/mg	1 h U/mg	24 h mM red. ends	24 h mM/AX%	10 min U/mg	1 h U/mg	24 h mM red. ends	24 h mM/AX%
WAX	17.0 ± 0.3	7.7 ± 2.1	16.7 ± 0.6	17.6 ± 0.6	22.8 ± 0.5	9.4 ± 0.4	16.9 ± 0.3	17.8 ± 0.3
RAX	25.0 ± 1.9	9.4 ± 1.9	14.8 ± 0.7	16.4 ± 0.7	34.3 ± 1.4	11.0 ± 0.7	15.3 ± 0.5	17.0 ± 0.5
BX	NA	NA	NA	NA	NA	NA	NA	NA
oat BG	NA	NA	NA	NA	NA	NA	NA	NA
OBF	1.6 ± 0.0	0.6 ± 0.3	0.7 ± 0.0	13.0 ± 0.2	0.8 ± 0.1	0.2 ± 0.2	0.9 ± 0.2	16.7 ± 3.7

AX = arabinoxylan; WAX = wheat arabinoxylan; RAX = rye arabinoxylan; BX = beechwood xylan; BG = oat β-glucan; OBF = alkali extracted oat bran fibers. NA = no activity. U = μmol/min.

### Enzyme activity optimum on wheat arabinoxylan

**Fig. 2:**
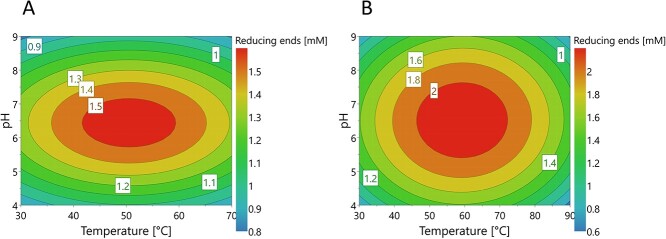
Response surface plots from optimization of reducing end formation by varying temperature and pH for a reaction using enzyme (**A**) *Hh*Xyn5A and (**B**) *Ct*Xyn5A on wheat AX. Response models and plots were generated using MODDE 12.1.

The most suitable reaction conditions regarding pH and temperature for a 10 min reaction on wheat arabinoxylan (WAX) were investigated for both *Hh*Xyn5A and *Ct*Xyn5A using a full factorial design experimental set-up, where the two factors were varied simultaneously. The *Hh*Xyn5A-CAT variant did not demonstrate detectable activity and was hence not possible to evaluate in this regard. The models created from the experimental data had high validity scores; *R*^2^ = 0.82 and *Q*^2^ = 0.68 for *Hh*Xyn5A, *R*^2^ = 0.79 and *Q*^2^ = 0.61 for *Ct*Xyn5A. The resulting response surface plots (Figure 2) imply that pH and temperature are non-correlated factors for activity, determined as reducing end formation by the DNS assay. The model also suggests that for maximal activity over a 10 min reaction on WAX, a pH around 6.5 should be used for both enzymes, whereas the temperature resulting in highest activity is around 50°C for *Hh*Xyn5A and 60°C for *Ct*Xyn5A. These optimum conditions were validated for both enzymes (data not shown) and were thereafter used when performing further experiments.

### *Hh*Xyn5A thermostability and irreversible deactivation

**Fig. 3:**
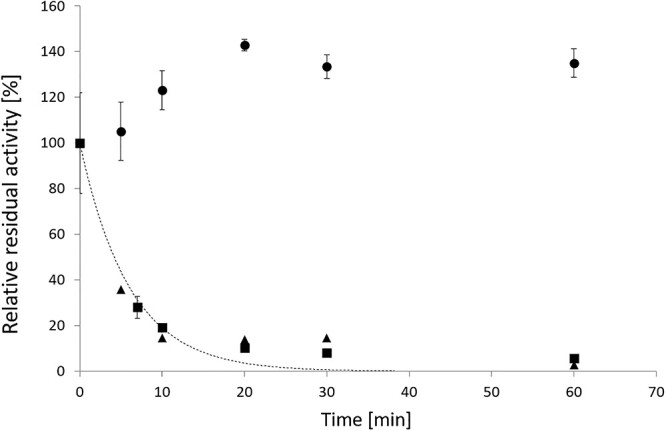
Deactivation curve of *Hh*Xyn5A after incubation for 60 min at 50°C (●) and 60°C (■), as well as for *Hh*Xyn5A in *Ct*Xyn5A formulation buffer at 60°C (▲). An exponential equation (dotted line) was fitted to the data for *Hh*Xyn5A at 60°C (■) to estimate the deactivation coefficient. After 4.15 min, only 50 percent of the activity remains. Each data point represents the mean value from duplicates or triplicates.

To further investigate the stability of *Hh*Xyn5A, the melting temperature (*T*_m_) and activity loss over time (irreversible deactivation) were studied. The nanoscale differential scanning fluorometry (nanoDSF) derived *T*_m_ for *Hh*Xyn5A and *Ct*Xyn5A (Table 1) show protein unfolding around 61 and 73°C, respectively, at pH 6.5. The sole catalytic domain construct *Hh*Xyn5A-CAT displayed a higher *T*_m_ than the two-domain variant *Hh*Xyn5A. Addition of the WAX substrate resulted in an increase in *T*_m_ of up to 3°C for *Hh*Xyn5A (Table 1), indicating that the enzyme substrate interactions stabilized the structure. Irreversible deactivation studies (without substrate) showed that at its *T*_m_ 61°C at pH 6.5, *Hh*Xyn5A lost 50 percent activity already after 4 min (Figure 3) while being stable at 50°C for more than 36 h. This coincides with the boundaries of the optimization model and supports the chosen reaction conditions. There was no difference in either activity or stability, when *Hh*Xyn5A was eluted in the enzyme formulation buffer or in the *Ct*Xyn5A formulation buffer (data not shown). This shows that the addition of imidazole, glycerol, CaCl_2_ and NaCl did neither affect the stability nor the activity of *Hh*Xyn5A under these reaction conditions.

### Enzyme activity on different substrates

Substrate utilization was studied using commercially available xylan fibers from rye (RAX) and WAX, as well as in-house alkali extracted oat bran fibers (OBF). *Hh*Xyn5A and *Ct*Xyn5A demonstrated comparable activities on OBF and the commercial cereal arabinoxylans from rye and wheat (Table 2). None of the enzymes hydrolyzed beech wood xylan (BX) or oat β-glucan (BG), indicating high substrate specificity and no or very limited β-glucanase side-activity. *Hh*Xyn5A-CAT showed no detectable activity on any of the substrates tested, suggesting that the CBM6 is necessary for catalytic function.

**Fig. 4:**
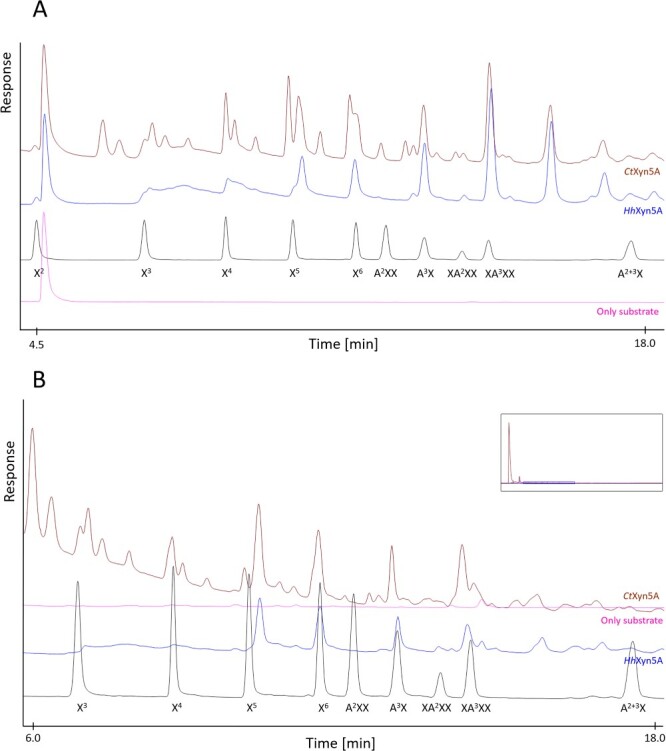
HPAEC-PAD chromatograms of oligosaccharide product profiles from enzymes *Hh*Xyn5A (blue) and *Ct*Xyn5A (brown) after 24 h using substrates (**A**) wheat arabinoxylan and (**B**) OBFs. The chromatograms were zoomed in and the first few minutes of the elution profiles were omitted, in order to visualize hydrolysis products of interest. Arabinoxylo-oligosaccharide standards are represented in the black curve of the chromatogram. A = arabinose; X = xylose.

The high performance anion exchange chromatography with pulsed amperometric detector (HPAEC–PAD) chromatograms show that *Hh*Xyn5A produced a quite different oligosaccharide profile pattern than *Ct*Xyn5A, after enzymatic reaction for 24 h on WAX and OBF (Figure 4). Both enzymes produced product peaks appearing at the same retention time as standards A^3^X and XA^3^XX, as well as a pattern of oligosaccharides with longer retention times. However, *Ct*Xyn5A produced additional shorter quantifiable XOS products, for example peaks eluting at the same retention times as X^4^ to X^6^, as well as multiple unknown oligosaccharides appearing at shorter retention times, some of which showed corresponding retention times to maltooligosaccharides (standards not shown).

### Alignment of GH5_34 enzymes

**Fig. 5:**
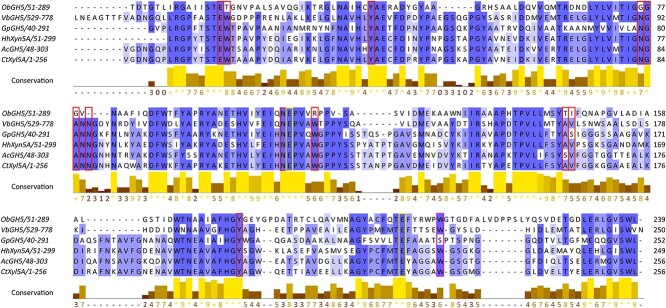
Multiple sequence alignment of catalytic GH5 domain of HhXyn5A and the GH5_34 enzymes indexed in the CAZy database. The domain location in the full enzyme amino acid sequence is indicated after the enzyme name. Dark blue columns represent residues with high percentage identity, whereas light blue represents a lower percentage identity. The columns of residues with important function or ligand interaction for CtXyn5A and HhXyn5A are highlighted with red and the catalytic residues are highlighted in yellow. Tryptophan residue (W) present in the amino acid sequence of CtXyn5A but not included in the crystal structure model (PDB 2Y8K and PDB 5LA2) is highlighted in the purple column. The figure is avaliable in color in the online version.

**Fig. 6:**
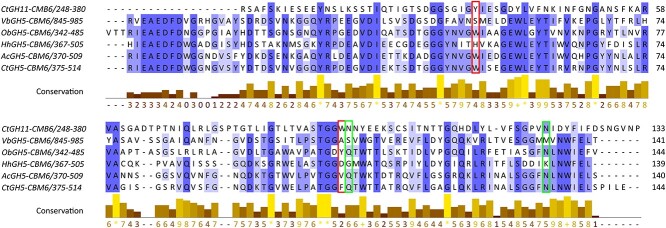
Multiple sequence alignment of the CBM6 domain of *Hh*Xyn5A and the GH5_34 enzymes indexed in the CAZy database, as well as *Ct*GH11-CBM6. The domain location in the full enzyme amino acid sequence is indicated after the enzyme name. Dark blue columns represent residues with high percentage identity, whereas light blue represents a lower percentage identity. The columns containing important residues for ligand binding in *Ct*GH11-CBM6 are highlighted in red for aromatic residues and in green for residues forming hydrogen interactions. The figure is avaliable in color in the online version.

The GH5_34 enzymes currently indexed in the CAZy database were compared with the novel *Hh*Xyn5A using a multiple sequence alignment of their catalytic GH5 modules (Figure 5) and CBM6 (Figure 6). For the catalytic GH5 modules, the two glutamic acid catalytic residues conserved within the family (Figure 5, yellow columns) are present in *Hh*Xyn5A, as well as other residues involved in hydrogen bonding or hydrophobic interactions of AXOS for *Ct*Xyn5A (Figure 5, red columns), for example, residues Glu68, Tyr92 and Asn139, which are important for arabinose interaction in the −2^*^ subsite (Labourel et al. 2016). Additionally, the GH5_34 enzymes from *Verrucomicrobiae bacterium* (*Vb*GH5), *Gonapodya prolifera* (*Gp*GH5) and *Acetivibrio cellulolyticus* (*Ac*GH5) from the CAZy database all have similar residue conservation and have all been shown to hydrolyze commercial arabinoxylans from wheat, rye and corn, displaying similar (A)XOS product pattern to *Ct*Xyn5A (Labourel et al. 2016). It should be noted that the tryptophan residue present in the amino acid sequence in both *Ct*Xyn5A and *Hh*Xyn5A (Figure 5*,* purple column), is missing in the tertiary crystal structure of *Ct*Xyn5A (PDB 2Y8K and PDB 5LA2), instead presenting gaps in this area.

A multiple sequence alignment was additionally generated for the CBM6, found as auxiliary modules of the GH5_34 enzymes, together with the well-characterized CBM6 from GH11 xylanase from *A*. *thermocellus* (initially *C. thermocellum*) (*Ct*GH11-CBM6) and the CBM6 from *Hh*Xyn5A (*Hh*GH5-CBM6) (Figure 6). The aromatic residues (red columns) and hydrogen binding residues (green columns) that have previously been identified as important residues for XOS binding in CBM6 in general (Charnock et al. 2000) and for *Ct*GH11-CBM6 specifically (Czjzek et al. 2001; Pires et al. 2004) are not conserved. Further investigation of the structural implications of these amino acid changes was performed using homology modeling and docking studies (section 2.6).

### *Hh*Xyn5A homology modeling and ligand docking

**Fig. 7:**
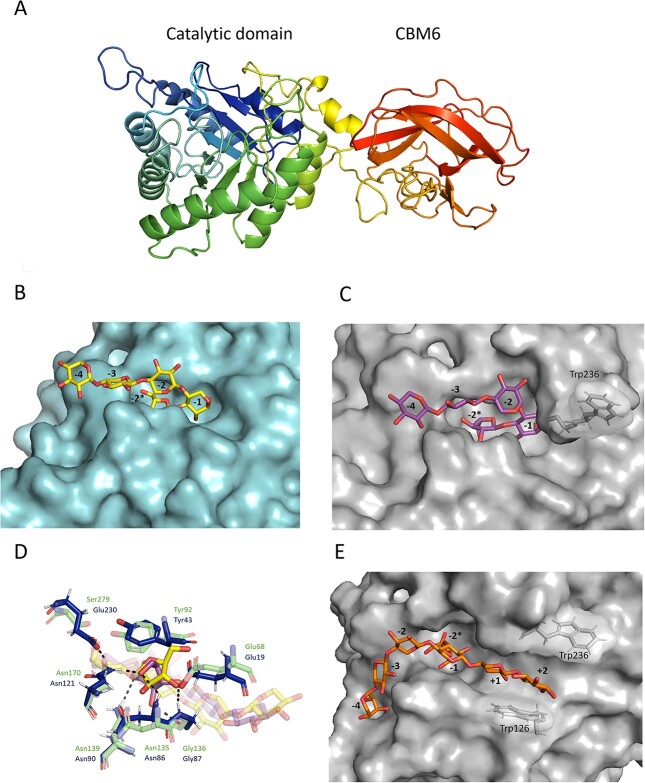
Comparison of arabinoxylo-oligosaccharide ligand docking in the active site of *Hh*Xyn5A and *Ct*Xyn5A. (**A**) Homology model of *Hh*Xyn5A. (**B**) Surface representation of *Ct*Xyn5A (blue) with XXXA^3^ ligand (yellow stick) based on crystallographic complex (PDB 5LA2). (**C**) Surface representation of homology model *Hh*Xyn5A (gray) with XXXA^3^ ligand (purple stick) based on docking and molecular dynamics simulations. Residue Trp236 is marked in the figure, as it is responsible for the extended loop region and to indicate that it is missing in the *Ct*Xyn5A tertiary structure but, however, present in its amino acid sequence. (**D**) Active site residues involved in ligand binding for *Hh*Xyn5A (blue stick) to XXXA^3^ (purple stick), overlaying *Ct*Xyn5A active site (green stick) and ligand (yellow stick) for comparison. The *Ct*Xyn5A structure used here is an inactive mutant (E279S). The *Hh*Xyn5A residues forming hydrogen bonding (black dashed lines) with the ligand in the −2^*^ subsite correspond to the residues forming hydrogen bonding in the *Ct*Xyn5A-ligand complex. (**E**) Surface representation of homology model *Hh*Xyn5A (gray) with an extended XXXA^3^XX ligand (orange stick) based on docking and molecular dynamics simulations. Residue Trp126 makes aromatic stacking interaction with xylose sugar in the +2 subsite. The figure is avaliable in color in the online version.

**Fig. 8:**
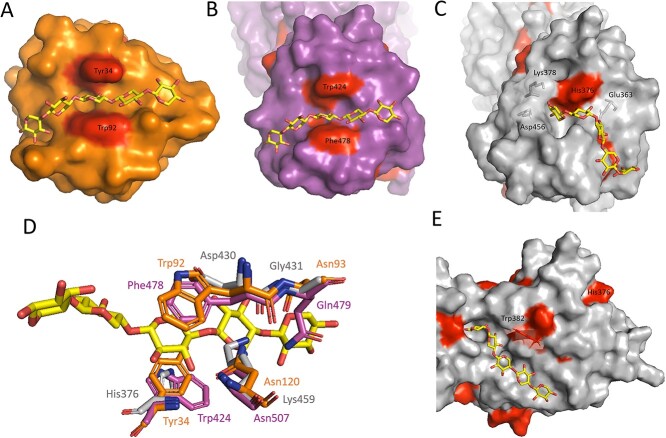
Ligand binding of xylooligosaccharide X^5^ in CBM6. (**A**) Crystallographic complex *Ct*GH11-CBM6 with ligand X^5^ in cleft A (PDB 1UXX). (**B**) Overlay of X^5^ into cleft A of *Ct*GH5-CBM6. (**C**) Docking simulation result of X^5^ close to cleft A of *Hh*GH5-CBM6. (**D**) Superimposition of *Ct*GH11-CBM6 residues (orange) in cleft A interacting with ligand X5 (yellow) and the structurally corresponding residues for *Hh*GH5-CBM6 (gray) and *Ct*GH5-CBM6 (magenta). (**E**) Docking simulation result of X^5^ close to cleft B site of *Hh*GH5-CBM6. Aromatic residues are colored in red on the surface representation images. The figure is avaliable in color in the online version.

In order to investigate the structure of *Hh*Xyn5A and compare it to the structures of *Ct*Xyn5A and *Ct*GH11-CBM6, a homology model was created and used for docking simulations with three ligands: XXA^3^ and XXXA^3^XX in the active site of the catalytic GH5 module (Figure 7C–E), and X^5^ in the potential binding clefts of the CBM6 (Figure 8C–E).

The *Hh*Xyn5A homology model displayed a tertiary fold and active site structure very similar to the *Ct*Xyn5A crystal structure. Docking simulations using XXXA^3^ as ligand further revealed a ligand fit comparable to previous crystallographic complex and simulations presented for *Ct*Xyn5A (Figure 7) (Brás et al. 2011; Correia et al. 2011; Labourel et al. 2016; Falck et al. 2018). The Xyl*p*-α-1,3-Ara*f* unit of the ligand binds in a pocket containing the −2^*^ and − 1 subsites, whereas the other Xyl*p* sugars make weak limited interaction with the enzyme (Figure 7B), similar to what has previously been suggested for *Ct*Xyn5A (Figure 7B) (Labourel et al. 2016). The modeled *Hh*Xyn5A-ligand complex formed hydrogen bonding from Glu19, Asn86, Gly87, Asn90 and Asn121 to arabinose in the −2^*^ subsite and xylose in the −1 subsite, which correspond to the hydrogen bonding shown for *Ct*Xyn5A residues Glu68, Asn135, Gly136, Asn139 and Asn170 (Figure 7D). In addition, the hydroxyl groups of the −2 to −4 Xyl*p* residues are pointing outwards into the solvent for both enzymes, indicating that a substrate with multiple arabinose substitutions could also be accommodated.

The residue Trp236 of *Hh*Xyn5A, responsible for the bulky loop region close to the active site (Figure 7C), is also present in the amino acid sequence of *Ct*Xyn5A (Figure 5*,* purple column). However, the tertiary crystal structure is missing parts of the residues in this region, instead presenting gaps and therefore making the loop adopt a smaller form (Figure 7B), compared with *Hh*Xyn5A. The longer ligand XXXA^3^XX can nevertheless be accommodated in the active site and shows aromatic stacking interaction with Trp126 in the +2 subsite (Figure 7E).

In contrast to the catalytic domain, the CBM6 for the two enzymes are quite different, structurally and in amino acid sequence. Previous studies on CBM6, including a study on *Ct*GH11-CBM6, have suggested that XOS substrates primarily bind in a region termed cleft A, located in the loop region between the two β-sheets of the classical jelly roll fold (Figure 8A–B). The hydrophobic interactions and stacking between the ligand sugars and aromatic residues in this cleft been have shown to be essential for substrate binding and specificity (Charnock et al. 2000; Czjzek et al. 2001; Pires et al. 2004). As previously seen in the multiple sequence alignment, *Hh*GH5-CBM6 is missing the important aromatic residues for X^5^ stacking present in *Ct*GH11-CBM6 and the CBM6 of *Ct*Xyn5A (*Ct*GH5-CBM6) (Figure 8C–D). The *Ct*GH11-CBM6 aromatic residues Trp92 and Tyr34, corresponding to Phe478 and Trp424 in *Ct*GH5-CBM6, are replaced with the acidic Asp430 and basic His376 in *Hh*GH5-CBM6 (Figure 8D). Residue Asn120, important for hydrogen bonding of X^5^ in *Ct*GH11-CBM6 (Pires et al. 2004), is also replaced by a longer Lys459 residue in *Hh*GH5-CBM6, as well as residue Asn93, interacting with X^5^ at subsites 4 and 5, which is replaced by the smaller Gly431.

The amino acid differences in *Hh*GH5-CBM6 result in a surface alteration of cleft A compared to *Ct*GH11-CBM6 and *Ct*GH5-CBM6 (Figure 8A–C), as well as quite different ligand binding as suggested from docking simulations of X^5^ in *Hh*GH5-CBM6. The second Xyl*p* residue does form a stacking interaction with His376; however, the loss of the second aromatic residue Trp92 (*Ct*GH11-CBM6 numbering) and other interacting amino acids has made the well-defined cleft disappear (Figure 8C). For *Ct*GH5-CBM6, the differences in amino acid sequence compared to *Ct*GH11-CBM6 does not seem to result in tertiary structural changes in cleft A, suggesting that X^5^ will bind in a similar way as illustrated by the overlay of the ligand in the potential binding cleft between the two aromatic residues Phe478 and Trp424 (Figure 8B).

The CBM6 family displays another binding region, cleft B, located on the flat β-sheet surface, which, in contrast to cleft A, is reported to be specific for cellulose and glucan recognition (Charnock et al. 2000). When performing a global docking simulation of X^5^ on *Hh*GH5-CBM6, one of the binding results with lowest energy was presented close to cleft B, where Trp382 could potentially make a hydrophobic interaction with X^5^ (Figure 8E). However, the binding site is shallow and is missing a second aromatic residue for potential stacking. No alternative deep cleft region, corresponding to cleft A with two aromatic residues, seems to be available at the *Hh*GH5-CBM6 surface.

## Discussion

The novel GH5_34 arabinoxylanase *Hh*Xyn5A could be produced at high yields with long storage stability, which makes the enzyme a potential candidate for large scale production, as no domain splitting or aggregation was observed. In contrast, *Ct*Xyn5A formulation is dependent on imidazole to prevent enzyme aggregation and precipitation (Schmitz et al. 2022a). *Hh*Xyn5A also displayed high stability at optimal reaction conditions of 55°C and pH 6.5 (Table 1 and Figure 3). The observed temperature optimum for *Hh*Xyn5A between 40 and 60°C (Figure 2A) is to be expected, since *H. hemicellulosilytica* was isolated from a biogas reactor incubating at 55°C (Koeck et al. 2015). The higher *T*_m_ observed for the sole catalytic domain variant *Hh*Xyn5A-CAT indicates that unfolding is starting with the CBM6 or in the hinge region between the two domains. It can however not be excluded that some type of misfolding occurred prior to the nanoDSF analysis, as the *Hh*Xyn5A-CAT construct did not demonstrate detectable activity on all substrates tested.

*Hh*Xyn5A displayed comparable activity to *Ct*Xyn5A on WAX and RAX, and neither enzyme was active on BX. Thus, *Hh*Xyn5A has the requirement of an arabinose substituent for catalytic function, in accordance with the profile of *Ct*Xyn5A, that has previously shown to be unable to hydrolyze non-substituted linear xylooligosaccharides (Labourel et al. 2016), corroborating the importance of arabinose substituents to bind in the −2^*^ subsite of the active site in GH5_34 subfamily.

The peaks observed for both *Hh*Xyn5A and *Ct*Xyn5A at the retention time of the known standard A^3^X (Figure 4) is instead likely to correspond to XA^3^, with the Ara*f* residue on the reducing end Xyl*p*, which is a proven product of *Ct*Xyn5A (Correia et al. 2011). As the compounds in principle have identical structures, it is likely that A^3^X and XA^3^ have similar retention times. The peak eluting at the same retention time as standard XA^3^XX is unlikely to correspond to this product, as an Ara*f* residue is required in the −2^*^ subsite of the enzyme, at the non-reducing end of the ligand. Therefore, XXXA^3^ is a more likely product to result from *Hh*Xyn5A and *Ct*Xyn5A hydrolysis, and may display a similar retention time. However, the coelution of XA^3^XX and XXXA^3^ cannot be confirmed due to lack of standards.

No hydrolysis of BG was observed using *Hh*Xyn5A, which is a beneficial trait for selective processing of cereal arabinoxylan to oligosaccharides, keeping polymeric BG of a high molecular weight intact. Polymeric BG is a valuable and desired product due to its health benefits, for example, lowering blood glucose levels and cholesterol (Wang and Ellis 2014).

The difference in the product profile shown on OBF and WAX (Figure 4) for the two enzymes is an interesting and somewhat unexpected finding, considering that the overall active site structure of *Hh*Xyn5A is very similar to *Ct*Xyn5A, based on both sequence alignment (Figure 5) and tertiary structure modeling (Figure 7). The homology model of *Hh*Xyn5A and the docking simulations performed in the active site of the catalytic domain, confirmed conservation of the active site residues (Figure 5) and demonstrated the possibility of the short ligand XXXA^3^ to be accommodated in a similar way as presented for *Ct*Xyn5A (Figure 7B–D) (Labourel et al. 2016). Considering the discrepancy between the *Ct*Xyn5A amino acid sequence and its determined crystal structure in regards to the missing tryptophan residue (Trp175), *Hh*Xyn5A does also not present any obvious conformational differences to *Ct*Xyn5A in proximity to the active site that could potentially sterically hinder longer or more substituted AXOS, and this does not explain the heterogeneity in product formation between the enzymes. Although the placement of the affinity tag for IMAC purification differs between *Hh*Xyn5A (N-terminal placement) and *Ct*Xyn5A (C-terminal placement), this would likely not affect the activity or product profile of the enzymes as the affinity tag sequence are distantly located from the active site in the tertiary structure (Figure 7A). C-terminal affinity tag placement substantially reduced storage and integrity stability of *Hh*Xyn5A (data not shown). Docking of a longer ligand, XXXA^3^XX, to *Hh*Xyn5A resulted in comparable accommodation as seen in the docking simulation with *Ct*Xyn5A (Falck et al. 2018) and showed similar aromatic stacking interaction with Trp126 (Trp175 in *Ct*Xyn5A) in the +2 subsite (Figure 7D). Based on these docking results, it is likely that other factors have a greater influence on the difference in activity profile shown between the two enzymes than the GH5 active site architecture.

The non-conserved sequence corresponding to cleft A in *Hh*GH5-CBM6 (Figure 6) is likely a factor of importance for the activity profile. Cleft A has previously been identified as important for XOS binding in the CBM6 family, and differences in sequence conservation has been proposed as an explanation for the diverse binding specificity seen within this CBM family (Czjzek et al. 2001; Michel et al. 2009). In this case, the missing important residues in *Hh*GH5-CBM6 lead to an alteration of the tertiary structure, loss of the cleft and loss of potential for multiple stabilizing aromatic stacking interactions of ligands (Figure 8C–D). *Hh*GH5-CBM6 might therefore rely more on hydrogen bonding and polar interactions for binding its substrate, for example through hydrogen bonding with Glu363, similar to the interaction displayed between X^4^ and Glu20 for CBM6 of endoglucanase 5A from *Cellvibrio mixtus* (Pires et al. 2004). The more open cleft A structure of *Hh*GH5-CBM6 may allow binding of xylans with various types of substituents. On the other hand, residues Lys378 and Asp456 of *Hh*GH5-CBM6 could sterically hinder longer substrates than the X^5^ ligand used here, at the reducing end (Figure 8C).

Depending on the substitution pattern of the AX substrate, the *Hh*GH5-CBM6 structure may be less adapted to bind certain regions of the AX polymer than corresponding *Ct*GH5-CBM6, which could result in the additional end products seen on WAX and OBF using *Ct*Xyn5A as compared with *Hh*Xyn5A (Figure 4). Further analysis of this would however require structural data of *Hh*GH5-CBM6. Moreover, the oat fiber substrate is not a commercial product, and the remaining impurities, potentially containing other fibers or structures connected to the oat AX, may affect the enzymatic activity (Schmitz et al. 2022b). This could be another reason for the differing product patterns between the two enzymes, where the oat substrate might be inaccessible for cleft A in *Ct*GH5-CBM6, whereas *Hh*GH5-CBM6 structure might be more suitable for binding. This hypothesis is supported by previously seen effects of other CMB6 influencing the catalytic activity on more recalcitrant or insoluble substrates (Charnock et al. 2000).

Alternatively, the AX substrates might bind in a different location than cleft A in *Hh*GH5-CBM6, potentially close to cleft B as demonstrated by docking simulations (Figure 8E), albeit there is no clear cleft structure or potential for multiple aromatic stacking interactions in this region. Previous studies show that the secondary structure of the binding site is fundamental for substrate selectivity (Johnson et al. 1996; Simpson et al. 2000) and that a single mutation of the residues in the binding cleft of a xylan-binding CBM can change the orientation of other residues and may result in changed or decreased specificity and even loss of activity (Simpson et al. 2000; Czjzek et al. 2001). It is thus most likely that the considerable difference in CBM6 cleft A between *Hh*Xyn5A and *Ct*Xyn5A is an important factor responsible for the different product patterns resulting after the respective enzymes’ activities on WAX and OBF.

Differences seen in product profile and substrate specificity has previously been assigned to differing domain organization of GH5_34 enzymes, for example, additional CBMs with alternative specificity or other catalytic domains with functions that mediate serial degradation of complex materials or increase activity on insoluble substrates (Charnock et al. 2000; Labourel et al. 2016). It would therefore also be of interest to investigate how other CBMs and the various domains, not related to attachment to the cellulosome, of the full-length *Hh*Xyn5A enzyme can influence the stability, activity and substrate specificity, for example, for the direct application of the enzyme in industrial oat fractionation processes, in order to increase the yield of soluble fiber and prebiotic AXOS in novel oat products.

To summarize, in this study the first GH5_34 arabinoxylanase from *H. hemicellulosilytica* was extensively characterized regarding stability, substrate specificity, product profile and substrate interaction. The novel GH5_34 arabinoxylanase *Hh*Xyn5A can be produced in high yields in *Escherichia coli*, and is stable during storage after purification and retains its activity at processing temperatures for several days, making it an interesting candidate for applied commercial use. *Hh*Xyn5A displays activity on commercial AX substrates from various grains, comparable to the activity of the commercially available homolog *Ct*Xyn5A with no side activity on BG. In contrast to *Ct*Xyn5A, *Hh*Xyn5A gave a more specific oligosaccharide product profile when using WAX as substrate, with longer XOS products. In addition, *Hh*Xyn5A was shown to be efficient on commercial WAX and RAX, as well as extracted oat fibers, producing several AXOS compounds with potential prebiotic effect, previously shown to be consumed by a probiotic *Bifidobacterium* species (Bhattacharya et al. 2020). The similarities of the GH5 active sites and the dissimilarities of the CBM6 binding sites between the homologs *Hh*Xyn5A and *Ct*Xyn5A suggest that it is the difference in the substrate binding sites of the CBMs that results in different product profiles. Further structural studies are needed to elucidate whether the difference is due to steric hindrance in cleft A of *Hh*GH5-CBM6, or due to the presence of an alternative recognition site for AXOS in this enzyme.

## Material and methods

### Enzyme candidate selection and sequence modifications

The truncated two-domain sequence of GH5_34 arabinoxylanase *Ct*Xyn5A (RefSeq WP_003513669.1 (Wilson et al. 2013)) from *A. thermocellus* (initially *C. thermocellum*) was used as query to search for similar sequences by blastp in the NCBI non-redundant protein sequence database under default parameter values. A putative GH5_34 subfamily arabinoxylanase (GenBank NLC19267.1 (Campanaro et al. 2020)) from *Clostridiales* bacterium (initially *H. hemicellulosilytica*) with 67.4 percent sequence identity (98 percent coverage) to *Ct*Xyn5A was selected. The *GX757_08645* gene (GenBank JAAZDK010000174.1) encoding the full-length multi-modular enzyme *Hh*Xyn5A-FULL variant as well as two truncated sequence variants were cloned (Figure 1A); one consisted of the catalytic GH5 domain and CBM6 for simplicity termed *Hh*Xyn5A, and the other construct *Hh*Xyn5A-CAT, consisted of sole catalytic domain. All constructs were cloned with an N-terminal His_6_-tag encoding sequence. The commercial GH5 arabinoxylanase *Ct*Xyn5A (product number CZ00601, NZYTech) is also a truncated two-domain enzyme (analogous to *Hh*Xyn5A) produced in *E. coli*.

### Cloning, expression and purification of recombinant *Hh*Xyn5A variants

Sequences encoding *Hh*Xyn5A and *Hh*Xyn5A-CAT were vector-adapted maintaining the native codon landscape, synthesized and cloned (Bio-Cat, Germany) in pET-21b(+) vectors (Merck). Heterologous overexpression was performed in *E. coli* BL21(DE3) (Merck). The enzyme targets were expressed by initial cell cultivation in Lysogeny Broth (LB)–Lennox at 37°C with shaking at 200 rpm until OD_600_ = 0.6–0.8. Isopropyl β-D-galactopyranoside was then added to 0.2 mM concentration to induce expression at 30°C with shaking at 200 rpm for 4 h. The cells were harvested by centrifugation at 10,500 × g for 15 min at 4°C, washed and re-suspended in lysis buffer (50 mM HEPES–NaOH pH 7.4 (RT), 500 mM NaCl, 50 mM imidazole and 5 percent (v/v) glycerol). After lysis of the cells by sonication (0.5 cycle for 10 min at amplitude 60 percent) using a UP 400S homogenizer (Hielscher Ultrasonics), the obtained lysate was clarified by centrifugation at 26,800 × g for 30 min at 4°C and the supernatant was filtered through 0.22 μm pore size filters.

Purification of proteins from clarified lysate was performed applying IMAC, with Ni^2+^ serving as ligand, with an ÄKTA Start system (GE Healthcare Life Sciences) using a 1 mL HisTrap HP (7 × 25 mm) column (Cytiva). The protein with N-terminal affinity tag bound to column in lysis buffer were eluted with elution buffer (50 mM HEPES-NaOH pH 7.4 (RT), 500 mM NaCl, 500 mM imidazole and 10 percent (v/v) glycerol) after extensive wash with lysis buffer. The purified proteins were dialyzed at 4°C through 3500 Da MWCO regenerated cellulose membranes against formulation buffer (50 mM HEPES-NaOH pH 7.2 (RT)) at 1:5000 volume ratio. Dialyzed samples were filtered through 0.22 μm pore size filters. In parallel, aliquots of the *Hh*Xyn5A enzyme variants were also eluted with *Ct*Xyn5A formulation buffer as according to manufacturer’s product information (35 mM HEPES-NaOH pH 7.5 (RT), 200 mM imidazole, 750 mM NaCl, 3.5 mM CaCl_2_ and 25 percent (v/v) glycerol) and filtered through 0.22 μm pore size filters. The protein concentration was determined by measuring absorbance at 280 nm (A_280_ 1 = 1 mg/mL as well as considering the absorption coefficient) using BioSpec-nano spectrophotometer (Shimadzu). Purity and integrity of proteins were analyzed by visualization applying 4–15 percent glycine–SDS–PAGE (Bio-Rad).

### Enzyme activity assay

Enzyme activity was measured by monitoring reducing end formation from different substrates with the di-nitrosalicylic acid (DNS) assay (Miller 1959). One unit (U) of arabinoxylanase activity was defined as the amount of enzyme required to produce 1 μmol of D-xylose reducing sugar equivalents per min from 10 mg/mL WAX in 50 mM HEPES–HCl pH 6.5 (60°C) buffer. Substrates were suspended at 10 mg/mL in reaction buffer (10 mM HEPES–HCl pH 6.5 (RT)) and added to 4.5 mL glass vials at 9:1 volume ratio of substrate and enzyme preparation of 0.2 mg/mL. The reactions were incubated in thermoshakers at 50°C for *Hh*Xyn5A or *Hh*Xyn5A-CAT and 60°C for *Ct*Xyn5A, with shaking at 500 rpm for all reactions. Samples were collected after 10 min, 1 h and 24 h, to be used for DNS assay and oligosaccharide analysis. DNS reagent was added to samples at a 1:1 volume ratio to stop the reaction and the samples were then incubated at 100°C for 10 min and chilled on ice, before measuring absorbance at 540 nm using with Multiskan GO microplate spectrophotometer (Thermo Fisher Scientific). Samples collected for oligosaccharide analysis were boiled for 10 min, subsequently diluted and filtered through 0.2 μm pore size filters.

Substrates including WAX (low viscosity), RAX and oat BG (medium viscosity) were purchased from Megazyme, whereas BX was purchased from Sigma–Aldrich. Alkali soluble OBF were extracted (Schmitz et al. 2022b) from an insoluble oat fiber bran fraction obtained from oat processing, provided by Lantmännen Oats (Sweden) in 2020 from their production site oat mill in Kimstad (Sweden). In short, the insoluble OBFs were milled, resuspended in water to 50 g/L and destarched using α-amylase and amyloglucosidase after gelatinization at 70°C for 2 h at 40°C. After washing with water, cloth filtration and freeze drying, the fibers were resuspended in Milli-Q purity grade water to 100 g/L, sonicated (10 min at 35 kHz) using a UP 400S homogenizer and centrifuged at 3893 × *g* for 10 min. The collected fibers were further re-suspended in 5 M NaOH to 100 g/L (of initial fiber weight) and incubated at 60°C for 9 h with constant shaking. After centrifugation at 3893 × g for 10 min, the supernatant was neutralized to pH 5–6 using 37 percent (w/w) HCl and the alkali soluble fibers were then precipitated using four volumes of 99 percent (v/v) ethanol overnight at 4°C. Finally, the fibers recovered by centrifugation at 3893 × g for 5 min to remove the ethanol, washed with water and the extracted fibers were freeze dried. AX content of the oat fibers were determined through acid hydrolysis and quantification of arabinose and xylose through HPAEC–PAD as previously described (Schmitz et al. 2021). All AXOS substrates had a similar A/X ratio of 0.6.

### Temperature and pH optimization using experimental design

Optimal temperature and pH for a 10 min reaction of *Hh*Xyn5A and *Ct*Xyn5A on WAX was investigated with quadratic full-factorial experimental design at three levels using MODDE 12.1 (Sartorius Stedim Data Analytics). To set the experimental conditions, 10 mM Tris–HEPES–acetate buffer at pH 4, 6.5, and 9 was used together with temperatures 30, 58.1 and 70°C for *Hh*Xyn5A and 30, 60 and 90°C for *Ct*Xyn5A. The enzyme activity assay was performed in a micro scale incubating thermocycler with heat gradient mode. The results were then interpreted in MODDE to create a model for prediction of the best reaction conditions for each enzyme.

### Enzyme stability

Enzyme melting temperature (*T*_m_) was estimated at different pH ranging 4–9, as well as pH 6.5 with 1 percent (w/v) WAX, using nanoDSF applying a Prometheus NT.48 instrument (NanoTemper Technologies). Standard grade glass capillaries (NanoTemper Technologies) were filled with enzyme solution at a concentration of 0.1–0.2 mg/mL. Thermal unfolding was performed with a temperature gradient between 20 and 95°C at a 1°C/min ramp rate and adjusting excitation power to 60 percent. *T*_m_ was determined from the first derivative of the absorbance ratio 350/330 nm and was identified automatically by the instrument software PR.ThermControl (NanoTemper Technologies).

*Hh*Xyn5A thermostability and inactivation over time was investigated by incubating the enzyme at 50 and 60°C for 24 h while monitoring the retained reaction rate by removing aliquots of enzyme over time and immediately chilling aliquots on ice. Retained activity was then evaluated in a 10 min reaction using 10 mg/mL WAX at 50°C with the DNS assay performed in a micro scale incubating thermocycler with heat gradient mode measuring absorbance using with Multiskan GO microplate spectrophotometer.

### Analysis of AX hydrolysis oligosaccharide product profile

The oligosaccharide profile after enzymatic hydrolysis of different substrates was investigated applying HPAEC–PAD with Dionex ICS-5000 system (Thermo Fisher Scientific) using a Dionex CarboPac PA200 (250 × 3 mm, 5.5 μm) analytical and a guard (50 × 3 mm) columns (Thermo Fisher Scientific). The separation was performed using a constant mobile phase composition of 100 mM NaOH at 0.5 mL/min, and a linear gradient from 0 to 30 min of 0–120 mM sodium acetate and thereafter a constant concentration of 160 mM sodium acetate until 40 min. AXOS standards xylobiose (X^2^), xylotriose (X^3^), xylotetraose (X^4^), xylopentaose (X^5^), xylohexaose (X^6^), 3^2^-α-L-arabinofuranosylxylobiose (A^3^X), 2^3^-α-L-arabinofuranosyl-xylotriose (A^2^XX), 3^3^-α-L-arabinofuranosyl-xylotetraose (XA^3^XX), 2^3^-α-L-arabinofuranosyl-xylotetraose (XA^2^XX) and 2^3^,3^3^-di-α-L-arabinofuranosyl-xylotriose (A^2+3^XX) were purchased from Megazyme.

### Sequence alignment, homology modeling and docking simulations

In order to review *Hh*Xyn5A attribution to the GH5_34 subfamily and define the amino acid conservation, the catalytic domain as well as the CBM6 sequences of the non-redundant GH5_34 subfamily enzymes indexed in the CAZy database (Drula et al. 2021) were retrieved from NCBI GenBank, aligned with *Hh*Xyn5A using the ClustalW online multiple sequence alignment tool (Sievers et al. 2011) and visualized using Jalview (Waterhouse et al. 2009). Domain boundaries were determined through NCBI Conserved Domain Database 3.17 (Lu et al. 2019).

A homology model of *Hh*Xyn5A was created with SWISS-MODEL (Waterhouse et al. 2018), with a Global Model Quality Estimate score of 0.92 (scored from 0 to 1) and global QMEANDisCo value of 0.89 ± 0.05, using the truncated *Ct*Xyn5A as template (PDB 2Y8K). All tertiary structures were superimposed and structurally compared using PyMOL 2.5 (Schrödinger). Amino acid numbering corresponds to the respective enzyme tertiary structure numbering.

Two AXOS ligands were prepared for docking in the active site of *Hh*Xyn5A using the Xyl*p*-β-1,4-Xyl*p*-β-1,4-Xyl*p*-α-[α-1,3-Ara*f*]-β-1,4-Xyl*p* (XXXA^3^) ligand from the crystallographic complex of *Ct*Xyn5A mutant E279S (PDB 5LA2) (Labourel et al. 2016). The original XXXA^3^ ligand was used as a base and extended by adding two Xyl*p* units to construct a longer XXXA^3^XX ligand, similar to previous modeling with *Ct*Xyn5A (Falck et al. 2018), using the YASARA 21.12.19 building tool (YASARA Biosciences). The resulting XXXA^3^XX ligand was energetically minimized by running a molecular dynamics simulation for 5 ns at 298 K and pH 6.5 using the AMBER14 force field. For studying ligand binding in *Hh*GH5-CBM6, an X^5^ ligand from an existing crystallographic complex of CBM6 from GH11 xylanase from *A*. *thermocellus* with X^5^ (PDB 1UXX) was used (Pires et al. 2004).

Docking of the ligands was then performed using AutoDock (Morris et al. 1998) implemented in YASARA, using the default parameter values supplied and keeping the active site residues flexible throughout the docking simulation. The resulting enzyme-ligand complexes were refined using molecular dynamics simulations performed in YASARA using standard procedure of the software. The simulations were run for 5 ns using the AMBER14 force field for the solute, at 298 K and pH 6.5. The energy minimized structures with similar orientation to the crystallographic complexes were then used as final complexes and visualized in PyMOL. The resulting root-mean-square deviation plots for each molecular dynamics simulation are shown in Supplementary Figures S1–S3.

## Supplementary Material

Supplementary_material_cwac080

## Data Availability

The data underlying this article are available in the article and in its online supplementary material.

## Abbreviations

AX: arabinoxylan; AXOS: arabinoxylo-oligosaccharides; BG: β-glucan; BX: beech wood xylan; CBM: carbohydrate-binding module; DNS: di-nitrosalicylic acid; DSF: differential scanning fluorimetry; GH: glycoside hydrolase; HPAEC–PAD: high performance anion exchange chromatography with pulsed amperometric detector; IMAC: immobilized metal affinity chromatography; OBF: alkali soluble oat fibre; RAX: rye arabinoxylan; RMSD: root-mean-square deviation; WAX: wheat arabinoxylan; XOS: xylo-oligosaccharides
