# Supplementary material for: Novel thermostable GH5_34 arabinoxylanase with an atypical CBM6 displays activity on oat fiber xylan for prebiotic production
Source: Glycobiology. 2022 Dec 6;33(6):490–502. doi: 10.1093/glycob/cwac080 (PMC10284105; doi:10.1093/glycob/cwac080)
Supplement: Supplementary_material_cwac080 [file supplementary_material_cwac080.docx]

Supplementary material

RMSD plots from molecular dynamics simulations of *Hh*Xyl5A and different ligands. For each Figure, the following information applies: A) Solute RMSD from the starting structure. The plot shows RMSD of Calpha (RMSDCa, blue), backbone (RMSDBb, red) and all-heavy atom (RMSDAll, green). B) The RMSD of the ligand heavy atoms over time, giving information about the movement of the ligand in the binding site. C) The RMSD of the ligand atoms over time and summarizes the conformational changes of the ligand.

**A**
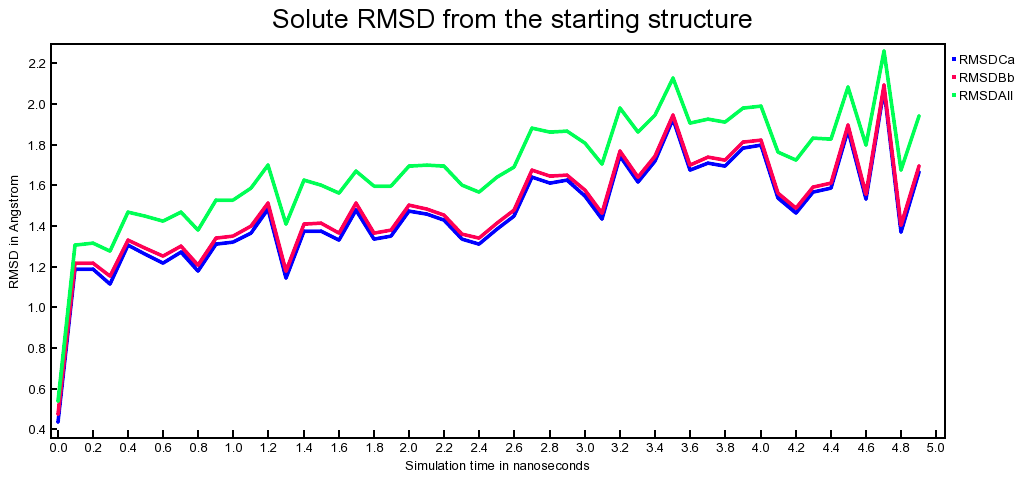


**B
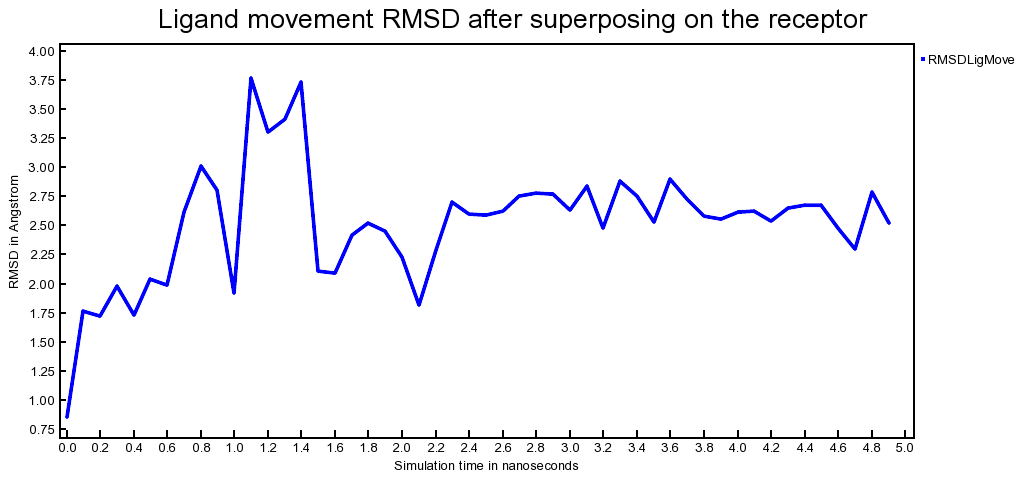
**

**C**
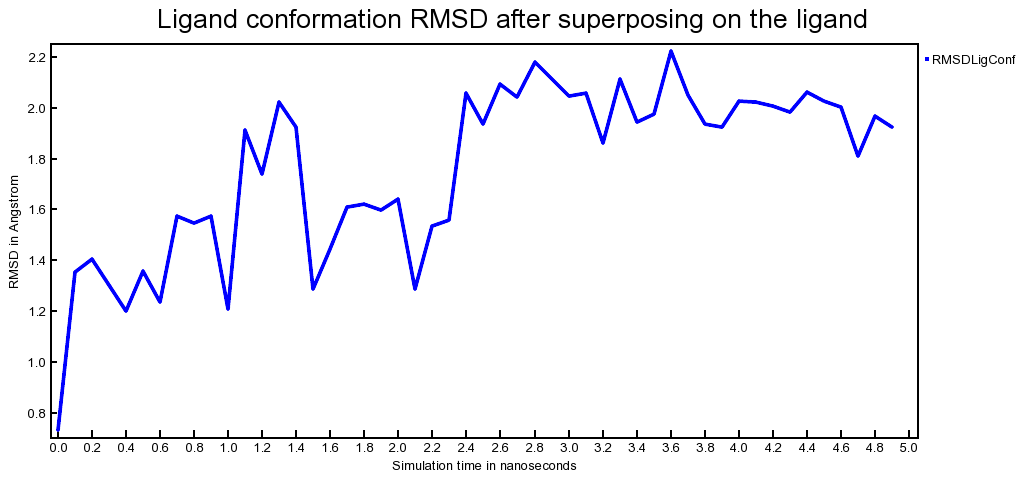


Figure S1. RMSD plots (A-C) from molecular dynamics simulation of *Hh*Xyl5A in complex with ligand XXXA^3^.

**A**
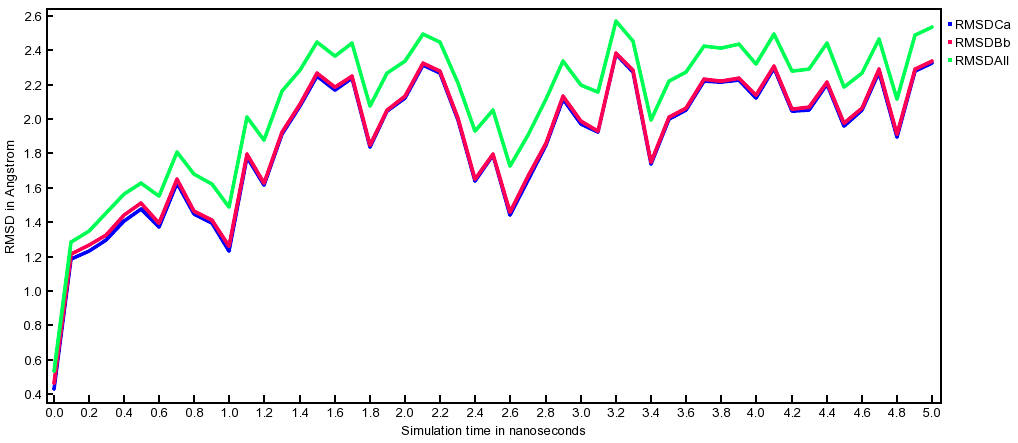


**B**
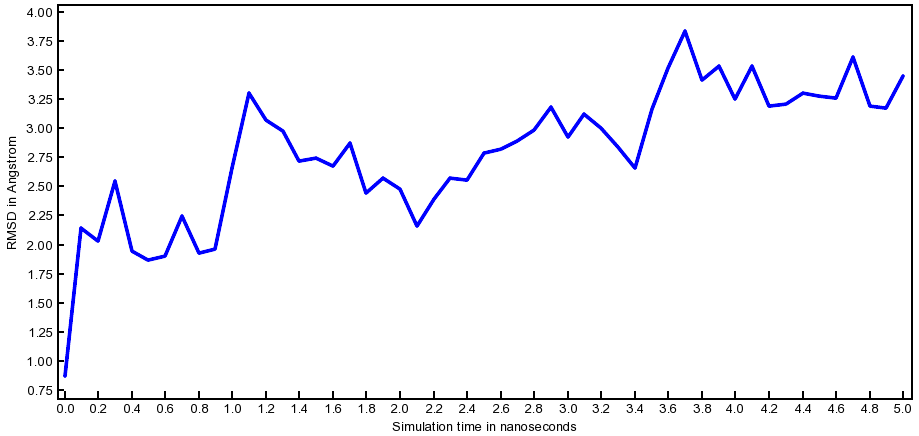


**C**
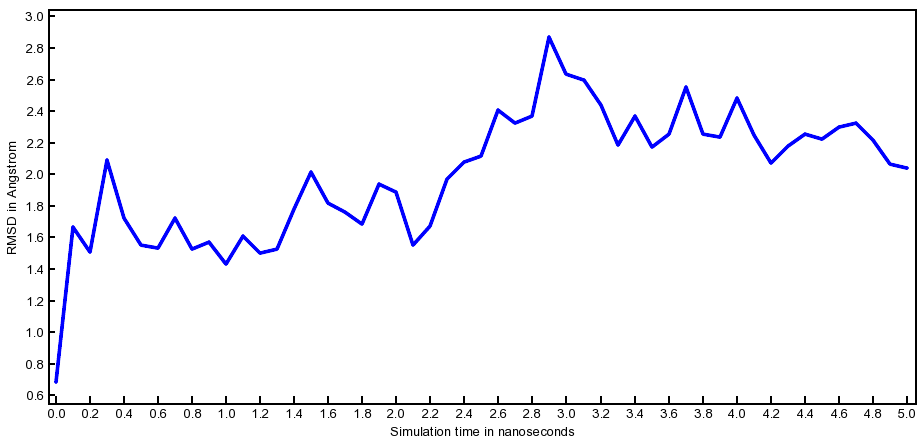


Figure S2. RMSD plot (A-C) from molecular dynamics simulation of *Hh*Xyl5A in complex with ligand XXXA^3^XX.

**A
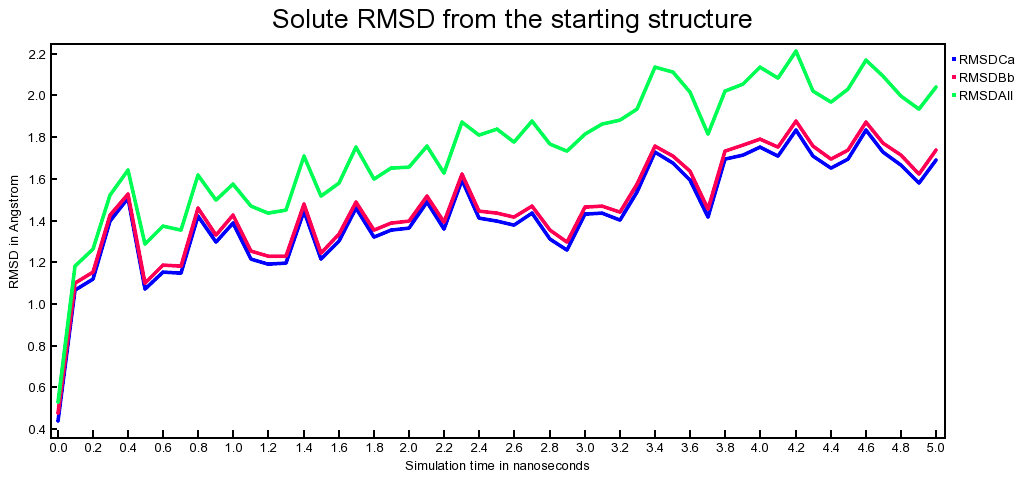
**

**B** **
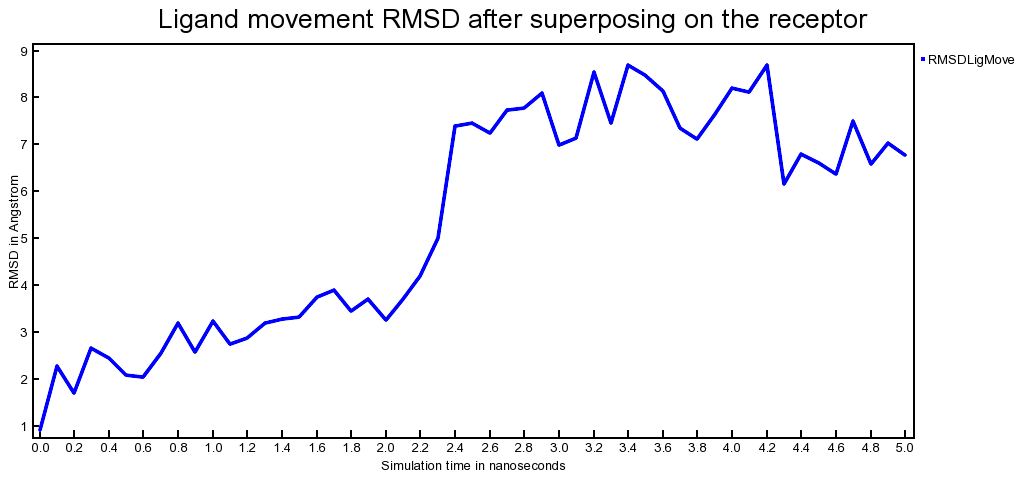
**

**C** **
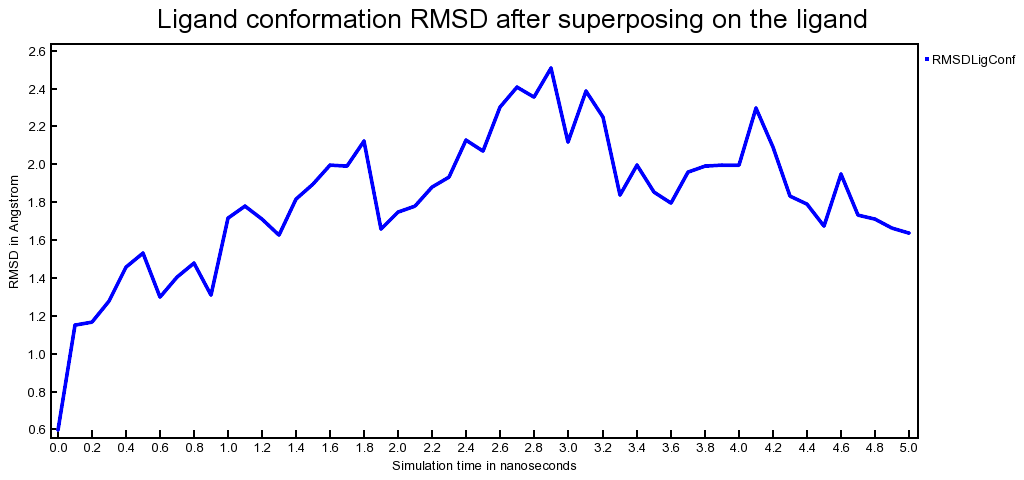
**

Figure S3. RMSD plot (A-C) from molecular dynamics simulation of *Hh*GH5A-CBM6 in complex with ligand X^5^.
